# Novel Piezoelectric Effect and Surface Plasmon Resonance-Based Elements for MEMS Applications

**DOI:** 10.3390/s140406910

**Published:** 2014-04-17

**Authors:** Sigita Ponelyte, Arvydas Palevicius

**Affiliations:** Kaunas University of Technology, International Studies Centre, A. Mickeviciaus g. 37, Kaunas LT-44244, Lithuania; E-Mail: arvydas.palevicius@ktu.lt

**Keywords:** piezoelectric effect, surface plasmon resonance, silver, tunable, periodical microstructure

## Abstract

This paper covers research on novel thin films with periodical microstructure—optical elements, exhibiting a combination of piezoelectric and surface plasmon resonance effects. The research results showed that incorporation of Ag nanoparticles in novel piezoelectric—plasmonic elements shift a dominating peak in the visible light spectrum. This optical window is essential in the design of optical elements for sensing systems. Novel optical elements can be tunable under defined bias and change its main grating parameters (depth and width) influencing the response of diffraction efficiencies. These elements allow opening new avenues in the design of more sensitive and multifunctional microdevices.

## 1. Introduction

Recently, a main challenge for researchers has been the design and manufacture of more efficient, reliable and accurate low-cost sensing devices. Surface Plasmon Resonance (SPR) of noble metal (such as gold, silver, *etc.*) nanoparticles has led to the development of many sensors with unique properties in the past few decades. The advantages of these plasmonic materials are significantly influenced by the distinctive optical properties of immobilized nanoparticles [1,2]. The immobilization of metal nanoparticles in a polymer matrix gives the sensing element the properties of processability and transparency. Using a combination of metal nanoparticles with polymers, it is possible to obtain exactly the right combination of electronic and optical properties which can be useful in solving problems such as a lack of sensitivity, accuracy, calibration, background signal, hysteresis, long-term stability, dynamic response, and biocompatibility [3]. Past researches have confirmed that novel nanomaterials with a high specific surface area (or surface-to-volume ratio) or high aspect to ratio (*i.e.*, length-to diameter ratio) can lead to a significant improvements in sensitivity and fast response time, exhibited by higher electron transport phenomena [4]. Application of nanotubes and nanofibers is one novel method for amplifying the sensing signal in immunosensors for medical purposes [5], acoustic and optical sensors used in detection of various solvents [6], novel optical pressure sensors in tactile robotic systems [7], imaging systems [8], *etc.* These sensors have had a huge impact in the food industry [9], bio-industry [10], medicine [11], environmental control [12], *etc.*, because of their capability to continuously and reversibly give a selective and fast response to the presence of a specific compound in a complex mixture of components, without perturbing the system.

The essence of piezoelectric sensors and biosensors is the piezoelectric effect. Piezoelectric sensors are mainly used in biosensing applications because of their simple construction and robust performance [13]. However, the small size and fragile nature of some piezoelectric materials leads to still very complicated sensing element designs. Sometimes using single materials in sensing can offer good spatial resolution, but due to their brittleness, the designed sensing elements are not reliable for robotics in medicine, military, automation, *etc.*, because of their rigid substrates, complex wiring or fragile elements. As for example, one of the major drawbacks of the incorporation of high aspect ratio nanomaterials, such as carbon nanotubes, into polymeric matrices is the increase of viscosity. To overcome the mentioned shortcomings, composite materials on a nanometric scale were proposed by many researchers. In this paper, a combination of specific materials (e.g., polyvinylidene fluoride (PVDF), lead zirconate titanate (PZT), Barium Titanate (BaTiO_3_), (poly(methylmethacrylate) (PMMA), *etc.*) and their properties help propose new ways for sensing devices to avoid certain limitations like fragility, size, form, sensitivity, reliability, rapid response, *etc.* Such films are more flexible and lightweight, with low acoustic impedance and a large working area compared to ceramics. Thus, the selection of suitable composite materials is one of main goals in the design of sensing elements for quantitative and qualitative analysis.

An undiscovered aspect of today's research on novel materials and elements is the surface modification possibilities in sensors for advanced applications. This paper discusses results obtained by implementing piezoelectric materials and periodical microstructure exhibiting SPR effects in a single novel deformable material—a tunable optical element. Electrical tuning has great advantages when compared to simple methods like tuning by cooling, heating or external pressure. The designed elements are rather simple to integrate in small microsystems and are low cost, simple and flexible. The selected materials and technological processes were chosen to be cost-effective (using low-cost microfabrication methods on a single substrate) so that the designed sensing system would be competitive in the economic sense compared to conventional methods able to reduce costs. The combination of specific properties in one element can significantly influence the results in analyte studies, medical, biomedical, optical or environmental applications. The tunable optical elements can be miniaturized, which is essential when designing e.g., communication applications (where beam switching is needed), displays with small pixels, monochromatic light sources or laboratory equipment for investigation of biological systems (when exposed to microstructure shape changing).

## 2. Experimental Section

### 2.1. Chemicals and Reagents

5% Polyvinylidene fluoride (PVDF, average M_W_ = 34,000) and nanoparticles of barium titanium oxide (BaTiO_3_) were taken in suitable and defined compositional ratios and mixed with 5% PMMA (average M_W_ = 15,000). Silver nitrate (AgNO_3_, analytical reagent) from Sigma Aldrich (Dorset, UK) was synthesized with PVDF-based mixture. All the reagents were of analytical grade and used as received without further purification. Deionised water was prepared with a Millipore water purification system. Conductive silver paste was used for formation of electrodes.

### 2.2. Formation of Elements

Thin films were produced on the pre-treated glass substrates. These substrates were sonicated for 10 min in acetone and chemically etched in warm special chrome solution (K_2_CrO_7_ + H_2_SO_4_ + H_2_O) for 10 min, and dried in an air stream. A spin-coating technique was used for processing thin film layers by means of a Dynapert Precima (Colchester, UK) centrifuge. Film of even thickness was obtained when the spin speed was 2,000 rpm and the centrifugation duration adjusted to 30 s. After spin coating, drying was performed in an oven at 90 °C for 10 min and then exposed by UV light using a Hg lamp (Philips, Aachen, Germany) of 1.2 kV (230–320 nm).

Periodical microstructures were fabricated onto thin films by low-cost and high-throughput replication technology—a hot embossing process. This process allows simultaneous and single-step formation of gratings on various microstructures with high accuracy. Thin film layer and master grating were placed together and fixed in the metallic mandrel between plates and putted in the furnace of 110 °C for 12 min (see Table 1.).

The hot embossing procedure in polymers was discussed in previous reports [14].

### 2.3. Design Concept

**Figure 1.**
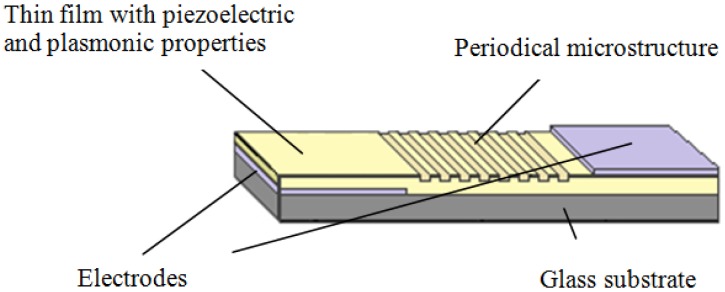
Active optical element based on piezoelectric and SPR effects.

The designed elements combine the properties of piezoelectricity and SPR. It is rather complicated in structure but simple in design (Figure 1). The development of novel elements includes three stages: (a) synthesis of biologically compatible materials into high aspect ratio thin films; (b) formation of electrode on substrate; (c) formation of thin films; (d) formation of gratings; and (e) formation of the electrode on top of the thin film. Thus, a layer with piezoelectric material is formed between an upper and lower electrode. By applying a voltage between the electrodes, they are attracted to each other by Coulomb forces. The periodical microstructure is implemented in the material such that the deformations (occurred when voltage is applied) influence periodic structure and the angle of diffraction of the grating. Two different type tunable optical piezoelectric–plasmonic elements (PVDF-PMMA-Ag and PVDF-PMMA-BaTiO_3_-Ag) were designed and the corresponding investigation results are further presented in this paper.

### 2.4. Analytical Equipment

The influence of silver nanoparticles in PVDF based solutions on the optical properties was confirmed by UV-VIS (UV/VIS/NIR AvaSpec-2048, Surrey, UK). The optical absorbance of the novel elements was measured by a spectrometer in the 200−700 nm wavelength range (accuracy of measurements 0.2%).

The morphology and elastic properties of novel thin films were investigated by atomic force microscopy (AFM) using a NT-206 instrument (Microtestmachines Co., Minsk, Belarus). A NSC11/15 V type non-contact silicon cantilever probe (Micromasch Inc., Wetzlar, Germany, force constant 3 N/m, resonant frequency 60 kHz) was used for measurements. Image processing and analysis of scanning probe microscopy data was performed using a Windows-based program Surface View 2.0. Statistical evaluation of the surface morphology was performed for a scanning area of 12 μm × 12 μm.

A PMT-3 (Okb Spectr, Saint-Petersburg, Russia) microhardness tester was used for evaluation of the formed thin film microhardness. It consists of a standard tester with integrated flat field optics and a main measuring tool—a diamond stylus probe. The substrate starts to contribute the measured hardness at penetration depths of the order 0.07–0.20 times the coating thickness [15] using five to seven loads ranging from 0.049 N. The indentation period was 15 s; 10 to 15 indentations were taken for each load. The coefficient of variation was not higher than ±8%. The Vickers diamond pyramidal indent diagonal was parallel to a prime flat for both orientations (*i.e.*, the diagonals were parallel with orientations for both substrate orientations). It was defined that the thickness of thin films on substrates was 10 times larger than the depth of the indentation. Thus, the influence of the substrate material was avoided. The average values of imprinted rhombus, *d* (in m) were calculated from independent measurements made for every load, *P* (expressed in N). The Vickers microhardness, *H_V_* (in GPa), was calculated using formula [15]:

(1) $$H_{V}=0.01854\cdot P\cdot d^{-2}$$

here, 0.01854 is a constant—a geometrical factor for the Vickers pyramid. The proportional specimen resistance (PSR) model of Li and Bradt [16] was used to evaluate and the variation of microhardness with loads. According to this model, the indentation test load *P* is related to indentation size *d* as follows [16]:

(2) $$P=a_{1}d+(P_{C}/d_{0}^{2})d^{2}$$

here, *P_c_* is the critical applied test load above which microhardness becomes load independent. Corresponding diagonal length of the indent is designated as *d*_0_. A plot of *P*/*d* against *d* gives a straight line. Obtained slope evaluates the load independent microhardness. The piezoelectric properties of novel piezoelectric–plasmonic elements were identified by vibration tests. A LabShop OFV–5000 Modular Vibrometer Controller (Polytec Ltd., Harpenden, UK) device was used to test the response of the elements at a defined bias.

## 3. Results and Discussion

PVDF polymer and BaTiO_3_ nanoparticles have been studied extensively, mainly in relation to their piezoelectric properties. Significant problem in the synthesis of novel element formation from piezoelectric materials is their miscibility with the amorphous phase, crystallization and the molecular origin of interactions. To overcome this limitation, piezoelectric materials were synthesized with the polymer PMMA. This is an original way to force PVDF to crystallize in the piezoelectric phase. The results also proved that no poling is needed for thin films when PMMA is introduced, *i.e.*, the piezoelectric elementary cells have a uniform orientation.

### 3.1. Evaluation of Optical Properties

**Figure 2.**
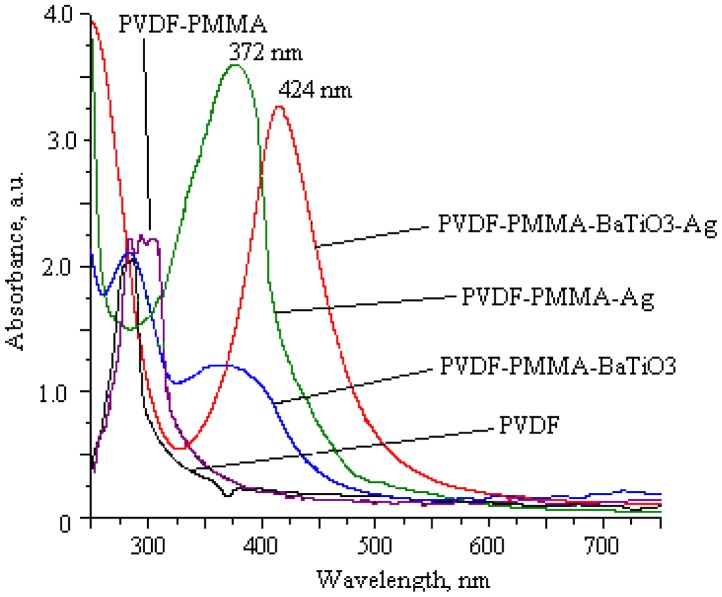
Comparison of absorbance spectra of PVDF-based films and films with silver nanoparticles.

The optical properties of PVDF-PMMA-Ag and PVDF-PMMA-BaTiO_3_-Ag were confirmed by UV-VIS. For better evaluation of how silver nanoparticles influence the optical response, novel thin films with Ag were compared with thin films fabricated under the same conditions but without Ag. Results are given in Figure 2. Here, the sharp absorption edge of pure PVDF were observed at a wavelength of 240 nm with absorbance of 2.1 a.u.; this peak indicates the semicrystalline nature of PVDF. A shift in band edges toward the higher wavelengths with different absorption intensity for PMMA synthesized with PVDF was observed at 290–320 nm with absorbance of 2.3 a.u. Adding to PVDF-PMMA some BaTiO_3_ leads to broadening of the peaks with a shift, *i.e.*, two dominating peaks are observed at 290 nm and 380 nm with absorbance of 2.1 a.u. and 1.25 a.u., respectively. Significant changes ocurred when PVDF-PMMA and PVDF-PMMA-BaTiO_3_ were synthesized with silver nanoparticles, *i.e.*, sharp dominating SPR peaks were observed at 372 nm with absorbance 3.6 a.u. for PVDF-PMMA-Ag and at 424 nm with absorbance 3.2 a.u. for PVDF-PMMA-BaTiO_3_-Ag. Therefore dominating peaks indicate the formation of inter/intra chain between PMMA and PVDF. Also, the shift in the absorption edge in the PVDF based films reflects the variation in the optical energy band gap.

Incorporation of Ag nanoparticles in piezoelectric films shifts a dominating peak in the visible light spectrum. Dominating peaks at 372 nm and 424 nm in Figure 2 are defined as SPR peaks and explained as silver nanoparticles agglomeration, *i.e.*, intensive formation of small silver particles in the polymer matrix [17]. Thus, the optical window is essential for design of active optical elements for sensing systems, because for most of them the working range is in the visible light spectrum from 400 to 700 nm. PVDF blends with Ag exhibit a well-defined window of wavelength range 372–424 nm. The diameter of the silver nanoparticles was approximately 50–60 nm.

### 3.2. Evaluation of Surface Morphology and Microhardness

**Figure 3.**
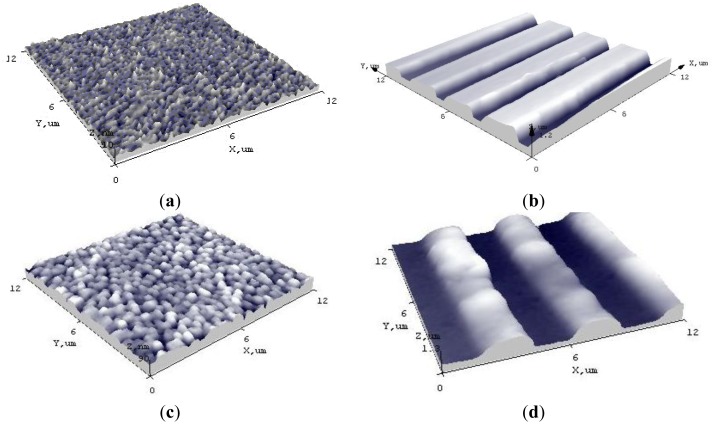
3D views of (**a**) PVDF-PMMA-Ag thin film and (**b**) grating on it; (**c**) PVDF-PMMA-BaTiO_3_-Ag thin film and (**d**) grating on it.

Surface morphology and elastic behavior of novel sensing elements with Ag were analyzed by atomic force microscopy. 3-D views of the surface morphology and grating on the novel PVDF-PMMA-Ag element is shown in Figure 3a,b. On its surface, very small islands are observed, *i.e.*, crystalline structures, with a surface roughness of 0.8 nm. There are irregularities of average depth of 3 nm and width of 0.6 nm. The large surface morphology parameters lead to a well defined regular periodical microstructure, imprinted onto the surface of PVDF-PMMA-Ag thin film (Figure 3b), *i.e.*, grating average width was 4 μm and average depth of 0.769 μm The element formed from PVDF-PMMA-BaTiO_3_-Ag has a rougher surface of about 8.1 nm, *i.e.*, BaTiO_3_ nanoparticles reduced the smoothness of the element (observed irregularities of average depth of 76 nm and width of 37 nm) (Figure 3c). Thus, the periodical microstructure imprinted onto the surface of the thin film was less similar to the master grating, *i.e.*, average depth of 0.73 μm with width of 3.9 μm (Figure 3d).

Previous research proved that the addition of PMMA to PVDF-based thin films significantly reduces the surface roughness of composite material and thin film becomes more elastic. The surface elastic behavior of novel elements was evaluated by load–distance curves (from AFM measurements). The contact between tip and thin film surface is elastic and no material transfer occurs at these loads when the adhesion forces, measured with increasing external loads, remain constant. Moreover, the degree of the roughness structures' deformation influences the adhesion of at the contact. This deformation is dominated by the attractive portion of the interacting forces between the film surface atoms of the contact tip. Thus, the adhesion values are one of the basic mechanisms of friction and also influence the deformation of thin films when periodical microstructures are imprinted. Measurement data is given in Table 2.

Microhardness measurements were performed by a PMT-3 microhardness tester, using five loads ranging from 0.049 to 0.392 N. The indentation period was 15 s; 10 to 15 indentations were taken for each load. The Vickers and absolute microhardness of the elements were evaluated. Thus, PVDF-PMMA-Ag have a rather elastic surface with Vickers microhardness of 0.647 ± 0.0026 GPa. Nanoparticles of BaTiO_3_ increase the microhardness for about 48% for PVDF-PMMA-BaTiO_3_-Ag element (Table 2). The PSR model was used for analysis in the variation of microhardness with loads, *i.e.*, calculating the load independent value—the absolute microhardness *H_A_* according to Formula (2). For the element PVDF-PMMA-Ag the absolute microhardness was 1.027 GPa, and for PVDF-PMMA-BaTiO_3_-Ag element it was almost three times larger (about 3.143 GPa). Thus, addition of BaTiO_3_ nanoparticles reduces the elasticity, but, at the same time, increases the microhardness of the elements' surface. The importance of mechanical properties evaluation is significant in many phenomena even beyond tribology, like coating performance, wettability, and micro/nanotechnology.

### 3.3. Evaluation of Piezoelectric Properties and Diffraction Efficiencies

**Figure 4.**
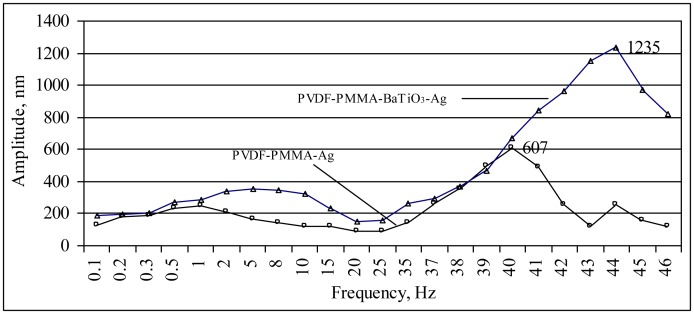
Amplitude—frequency dependence of the elements (voltage 130 mV).

There are a few ways to determine the piezoelectric effect and its value in a piezoelectric material. One way is to observe the converse piezoelectric effect by applying a certain voltage to the material. In this paper, the indirect piezoelectric effect of thin films was observed with a help of a vibrometer—a Pulse LabShop instrument. The drawback of this method—the observation of changed grating deformation is quite difficult, as the deformations occurring are very small, and a special high accuracy measuring devices has to be used. The amplitude—frequency dependence of the PVDF-PMMA-Ag and PVDF-PMMA-BaTiO_3_-Ag elements is given in Figure 4.

When an AC voltage of 130 mV (Figure 4) is applied to the piezoelectric thin film, varying the frequency it can be seen that there is a specific frequency at which the film starts to vibrate at higher amplitudes. This frequency, so called resonant frequency, for a PVDF-PMMA-Ag piezoelectric–plasmonic film was 40 Hz with a displacement of 607 nm, and for PVDF-PMMA-BaTiO_3_-Ag it was 44 Hz with a displacement of 1,235 nm. Thus, applying too high a voltage (higher than 310 mV), no response is registered and the elements stops oscillating, *i.e.*, the piezoelectric effect disappears. The designed elements are low frequency elements and this feature allows them to be integrated in systems where motion can harvest the energy, *i.e.*, wireless sensor networks or human monitoring devices where energy harvesting occurs from the human motion. The power density of such devices has a limited frequency range of less than 50 Hz [18,19] and design of the elements resonating at this frequency range with a large bandwidth, is a step towards novel solutions for low frequency monitoring devices.

**Figure 5.**
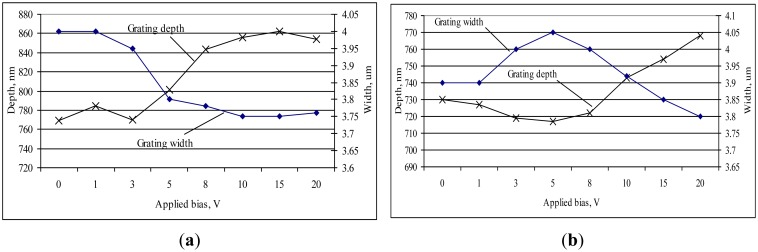
Changes of grating geometry under applied bias (**a**) PVDF-PMMA-Ag and (**b**) PVDF-PMMA-BaTiO_3_-Ag.

Further, the designed novel elements were investigated by applying a defined bias and observing the changes of the gratings' geometry parameters—grating width and its depth. Using AFM, the differences of microstructure relief to applied voltage were evaluated at each step and are given in Figure 5.

The obtained results showed that applying a voltage from 0 to 3 V gives only very small changes in the grating geometries of the elements: ∼12 nm in grating depth and ∼0.05 μm in grating width. Increasing the voltage up to 10 V, the grating width varies ∼30 nm and depth ∼0.1 μm in both optical elements. Thus, descriptive statistics (Table 3) of the experimental results show that PVDF-PMMA-Ag has a higher standard deviation indicating that the measured grating depth and width are spread out over a larger range of values than PVDF-PMMA-BaTiO_3_-Ag. The element has rather large positive linear association of grating width and depth on applied bias, too.

**Figure 6.**
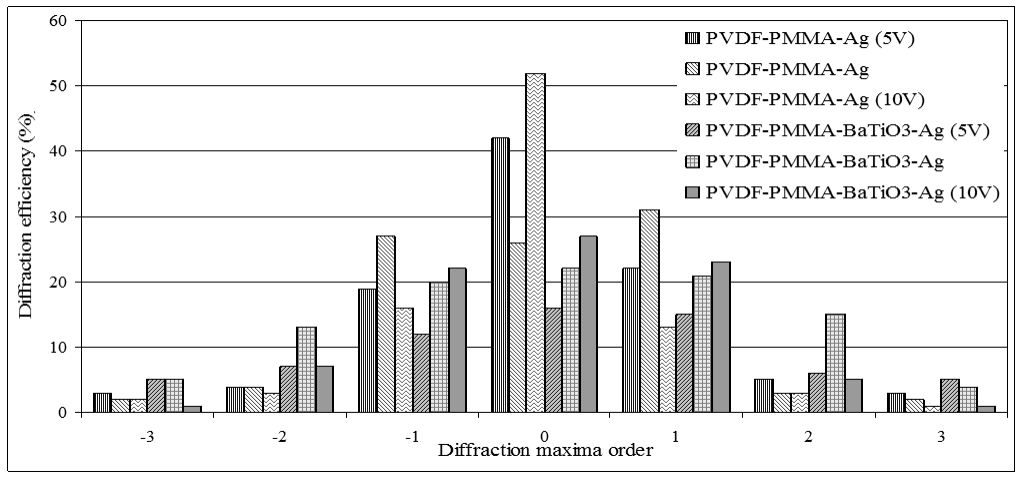
Diffraction efficiencies before and after applied bias to novel elements.

The results stated above prove the fact that when voltage is applied to a piezoelectric material, it stretches the grating changing the periodicity of the grating. It affects the angle of diffraction of the grating, too. These changes were registered by the diffractometer. Distribution of diffraction efficiency was observed in 0, ±1, ±2 and ±3 order maxima. Results showed that designed optical elements are based on a high efficiency reflection type gratings, *i.e.*, elements mainly diffracts a maximum amount of light into the first order maxima and then minimizes the amount of light in its zero and higher orders. Characteristics of efficiencies were registered and given in Figure 6.

Measurement results of the diffraction efficiencies of PVD-PMMA-Ag show that the applied bias significantly changes the efficiency distribution in different diffraction maximum orders, *i.e.*, its zero order efficiency may change from 15% to 30% depending on the applied bias of 5 V and 10 V, in its first orders about 8%–11%. Diffraction efficiencies of PVDF-PMMA-BaTiO_3_-Ag element can vary in its zero order about 10%, in its first orders about 7% when the voltage varies from 5 to 10 V. Results define that designed novel optical elements may be integrated in such applications where control over a light beam of the diffraction angle is needed.

Results prove the relevance of novel elements based on piezoelectric and surface plasmon resonance effect in a single chip. Simply said, the optical, mechanical, piezoelectric and surface properties become significantly important when dealing with composite materials used to construct smaller, faster, cheaper and more efficient optical elements, which can be routinely employed as active optical components in MEMS and MOEMS. Novel elements may be constructed for easy experimental monitoring of the element reaction, when the surface of the biosensor is in contact with a liquid analyte to be investigated. The integrated piezoelectricity and optics techniques in a single element is an excellent tool for the *in situ* study of the kinetics and equilibrium constants of relevant surface processes in biomedicine, medicine, pharmacy, *etc.*

## 4. Conclusions

The research results with the designed novel elements showed that surface plasmon resonance in piezoelectric–polymeric materials is not a typical phenomenon and implementation of metal (Ag) nanoparticles increase the absorbance for the specified wavelength. These tunable elements can be miniaturized and integrated in areas where beam switching is needed, in displays with small pixels, used as monochromatic light sources or for laboratory equipment to investigate biological systems. Low frequency working ranges of the elements is essential when designing human monitoring devices where the frequency limitations are defined. Moreover, it is possible to select appropriate piezoelectric and SPR effects-based elements, working at a wavelength range between 372 and 424 nm in order to increase the functionality and sensitivity of the overall system.

Results of optical elements with piezoelectric and SPR properties proved the relevance of voltage-driven periodical microstructures which change their geometrical parameters when the voltage is applied, *i.e.*, the average grating depth can vary by approximately 30 nm and the average width of the grating by about 100 nm.

These investigations open the gates to more significant challenges in the design of microsystems—the combination of SPR with piezoelectric effects for identification techniques, pushing the sensitivity towards the single-molecule detection limit, and, most importantly, practical development of sensing elements for routine use in humans' everyday life.

## Figures and Tables

**Table 1.** Technological data for formation of periodical microstructures.

**Master Grating Periodicity**	Depth	700 nm
	Periodicity	4 μm

**Master Grating Dimensions**	Length	2 mm
	Width	16 mm

**Grating Lines**	Parallel to the short edge	

**Pressure in Metallic Mandrel**	12 MPa	

**Table 2.** Mechanical properties of piezoelectric–plasmonic elements.

**PVDF-PMMA-Ag**	**PVDF–PMMA-BaTiO_3_-Ag**
The Vickers microhardness (Formula (1)): *H_V_* = 0.647 ± 0.0026 GPa	The Vickers microhardness (Formula (1)): *H_V_* = 1.223 ± 0.0029 GPa
The absolute microhardness (Formula (2)) *H_A_* = 1.027 GPa	The absolute microhardness (Formula (2)) *H_A_* = 3.143 GPa

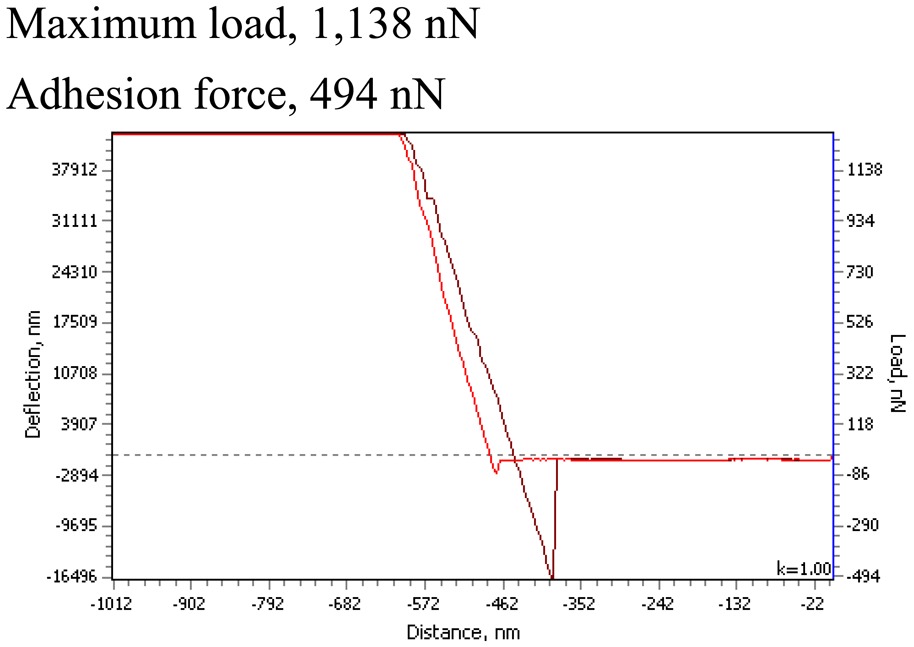	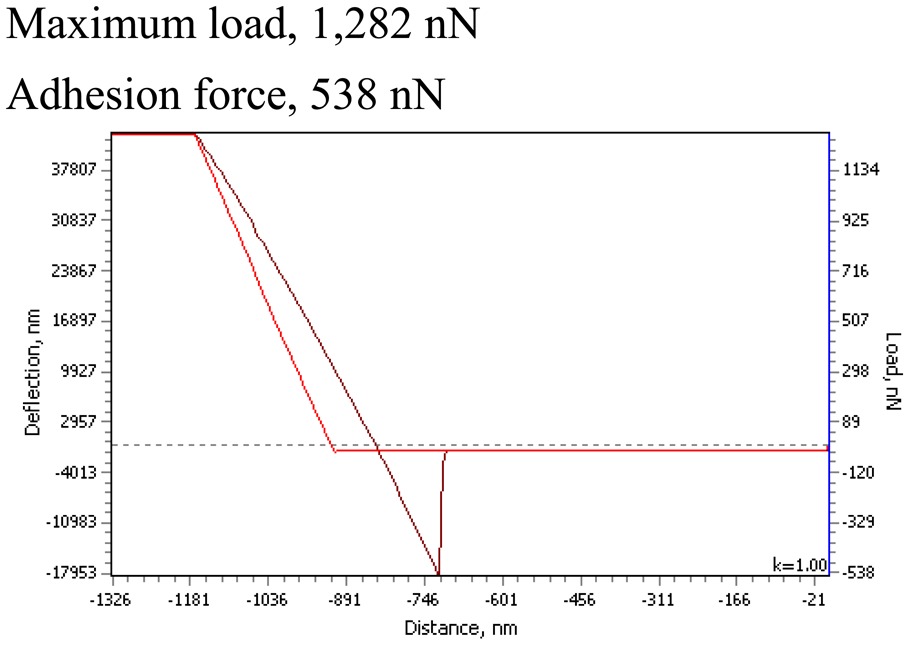

**Table 3.** Descriptive statistics of the grating geometry dependence on the applied bias.

**Descriptive Statistics**				

**N = 8 Measurements (0–20 V)**	**Element**	**Std. Deviation**	**Variance**	**R–Squared Value**
*Grating width, μm*	PVDF-PMMA-Ag	0.11370	0.013	0.676—large positive linear assoc.
	PVDF-PMMA-BaTiO_3_-Ag	0.08396	0.007	0.341—small positive linear ssoc.

*Grating depth, nm*	PVDF-PMMA-Ag	40.44021	1,635.411	0.766—large positive linear assoc.
	PVDF-PMMA-BaTiO_3_-Ag	18.33030	336.000	0.743—large positive linear assoc.
